# An Unusual Case of Seronegative Systemic Sclerosis and Cardiac Tamponade

**DOI:** 10.7759/cureus.89429

**Published:** 2025-08-05

**Authors:** Mohd Rafiw Ahmed Mahen, Rama Sami Issa Taha, Akil G Parmar, Beena Hameed

**Affiliations:** 1 Internal Medicine, King's College Hospital London, Dubai, ARE; 2 Internal Medicine, Mediclinic Hospital, Abu Dhabi, ARE; 3 Rheumatology, King's College Hospital London, Dubai, ARE

**Keywords:** diffuse cutaneous systemic sclerosis, limited cutaneous systemic sclerosis, nailfold capillaroscopy, pericardial effusion, seronegative, systemic sclerosis (ssc)

## Abstract

Systemic sclerosis (SSc) is an autoimmune rheumatic disease marked by excessive extracellular matrix deposition, causing fibrosis, endothelial dysfunction, and microvascular injury. There are two major types of SSc, limited and diffuse. SSc can affect any organ, leading to dysfunction and failure. The esophagus, kidneys, heart, and lungs are commonly affected. We report a case of a male patient who was diagnosed with scleroderma despite the absence of the classic antibodies and a subsequent presentation with cardiac involvement. This case highlights a rare presentation of seronegative SSc, emphasizing that diagnosis may extend beyond antibody testing. Additionally, it underscores the impact of cardiac involvement on patient morbidity and mortality.

## Introduction

Systemic sclerosis (SSc) is an autoimmune rheumatic disease marked by excessive extracellular matrix deposition, causing fibrosis, endothelial dysfunction, and microvascular injury. There are two major types of SSc, limited and diffuse [1]. The prevalence of SSc ranges between 50 and 300 million cases per one million people, and the incidence ranges from 2.3 to 22.8 cases per one million people per year [2]. It predominantly affects women (3:1 to 14:1 ratio), with a mean diagnosis age of 50 [1]. Scleroderma can affect any organ, leading to dysfunction and failure. The esophagus, kidneys, heart, and lungs are commonly affected [3]. We report a case of a male patient who was diagnosed with scleroderma despite the absence of the classic antibodies and a later presentation with cardiac involvement. This case highlights a rare presentation of seronegative SSc, emphasizing that diagnosis may extend beyond antibody testing. Additionally, it underscores the impact of cardiac involvement on patient morbidity and mortality.

## Case presentation

A 49-year-old man presented with swelling of his feet going up to his calves, morning stiffness that lasted for 3-4 hours, tightening of skin in both his upper limbs and lower limbs, and darkening of his hands and feet. His symptoms began six months ago and gradually progressed over time. His symptoms made it difficult for him to perform day-to-day tasks such as holding a cup of coffee. He did not report any rash, oropharyngeal ulcers, dysphagia, odynophagia, or changes in the color of his hands. Examination revealed limb stiffness, skin thickening, periungual hyperemia, and ankle joint tenderness. The clinical features were highly suggestive of scleroderma, limited subtype. All necessary investigations were done. Initial blood workup revealed normal cell counts, renal function, electrolytes, liver function, and C-reactive protein. Further work-up involved an antinuclear antibody comprehensive profile, which was negative for all antibodies; results are listed in Table 1.

**Table 1:** Antinuclear antibody comprehensive profile dsDNA: Anti-double stranded DNA; SS-A: Anti-Sjögren's syndrome-related antigen A antibody; Ro-52: anti-TRIM21 antibody; SS-B: anti-Sjögren's syndrome type B antibody; RNP/sm: anti-Smith/ribonucleoprotein antibodies; sm: anti-Smith antibodies; SCL-70: anti-topoisomerase I antibody; PML-SCL: polymyositis-scleroderma 100 antibody; Jo-1: anti-histidyl tRNA synthetase antibody; PCNA: anti-proliferating cell nuclear antigen antibody; AMA-M2: anti-mitochondrial M2 antibody.

Details	Value w/Units	Normal Range
dsDNA	0 IU/ml	<=8 IU/ml
Nucleosomes	0 IU/ml	<=8 IU/ml
Histones	4 IU/ml	<=8 IU/ml
SS-A	1 IU/ml	<=8 IU/ml
Ro-52	3 IU/ml	<=8 IU/ml
SS-B	2 IU/ml	<=8 IU/ml
RNP/Sm	1 IU/ml	<=8 IU/ml
Sm	1 IU/ml	<=8 IU/ml
Mi-2alpha	5 IU/ml	<=8 IU/ml
Ku	1 IU/ml	<=8 IU/ml
Centomere B	2 IU/ml	<=8 IU/ml
Scl-70	2 IU/ml	<=8 IU/ml
PM-Scl100	3 IU/ml	<=8 IU/ml
Jo - 1	2 IU/ml	<=8 IU/ml
Ribosomal (RIB)	0 IU/ml	<=8 IU/ml
PCNA	0 IU/ml	<=8 IU/ml
AMA-M2	1 IU/ml	<=8 IU/ml

**Figure 1:**
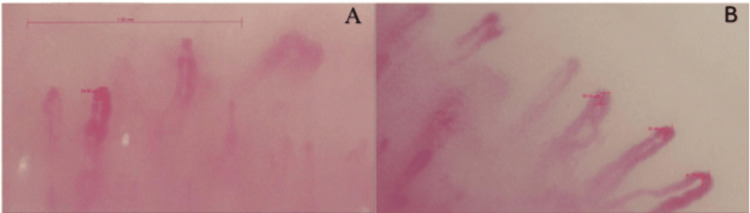
Nail bed capillaroscopy A and B show reduced capillary density, joined loops, hemorrhages, and neoangiogenesis features consistent with systemic sclerosis.

Spirometry was also normal. Despite the lack of biochemical findings, SSc was strongly suspected, and hence, capillaroscopy was done to investigate further. Nailfold capillaroscopy confirmed active scleroderma with reduced capillary density, joined loops, hemorrhages, and neoangiogenesis (Figures 1A, 1B).

**Figure 2:**
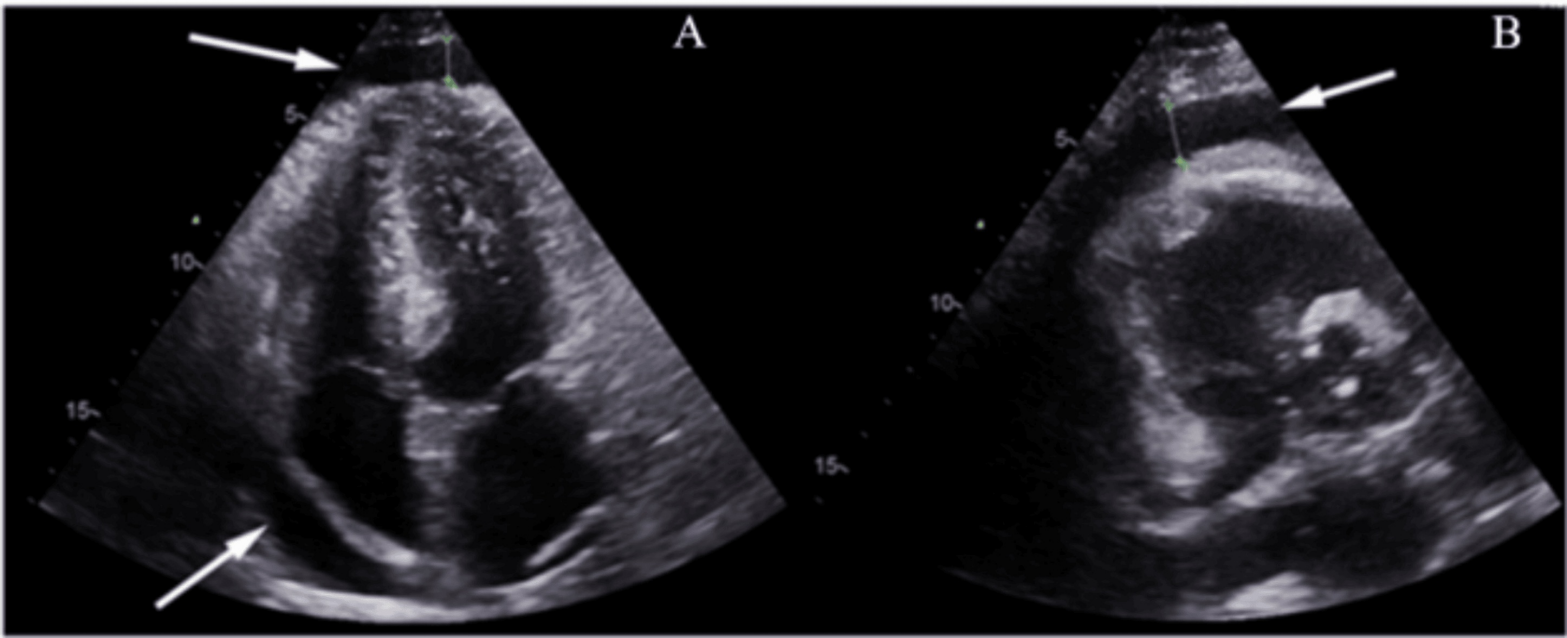
Transthoracic echocardiogram (TTE) A and B show a TTE demonstrating pericardial effusion. A: Apical four-chamber view showing a large anechoic space surrounding the heart, consistent with a circumferential pericardial effusion (white arrows). B: Parasternal long-axis view also revealing a significant pericardial effusion (white arrows), most prominent posteriorly.

With SSc now confirmed, the patient was started on treatment with corticosteroids and methotrexate. However, his symptoms persisted, prompting a switch to mycophenolate mofetil (MMF), which led to significant symptom relief and improved quality of life. The patient was doing well and was following up regularly. Two months later, he presented to the emergency department with complaints of lower limb swelling and chest heaviness. Symptoms were ongoing for two days and developed gradually. Initial workup revealed a normal ECG and troponin I, which were serially repeated. Other blood parameters revealed normal cell counts, elevated creatinine (1.90 mg/dL; reference range: 0.70 to 1.35 mg/dL), and a CRP of 49.60 mg/L (reference range: <5 mg/L). The autoantibody profile was repeated; however, this time the anti-RNA polymerase III antibody was positive, 97 units (normal <20 units). The patient’s symptoms persisted despite analgesia. The next troponin-I was positive; however, there were no ECG changes. Cardiology was consulted, and an urgent echocardiography was done. Echocardiography revealed a large pericardial effusion (Figure 2).

The patient deteriorated further, becoming hypotensive and tachycardic and requiring oxygen supplementation. An urgent pericardiocentesis was done, after which he gradually improved. The patient continued therapy with MMF and was discharged in a stable condition.

## Discussion

SSc is an autoimmune disease that is characterized by microvascular injury, mononuclear cell infiltration, and eventually fibrosis. Over time, it leads to loss of cells and atrophy [1]. There are two types of SSc, limited cutaneous SSc (LcSSc) and diffuse cutaneous SSc (DcSSc). Both types have skin involvement that commonly manifests as sclerosis; however, in LcSSc, skin involvement is distal compared to DcSSc, which affects the proximal limb and trunk. DcSSc is associated with early organ involvement affecting the lungs, kidneys, and heart [4]. Although Raynaud’s phenomenon (RP) occurs in approximately 95% of patients with SSc, our patient exhibited no signs of Raynaud’s disease. He initially had skin tightening, joint stiffness, and swelling of the lower and upper limbs distally, which are commonly associated with SSc [4].

Autoantibodies are a hallmark of SSc; antinuclear antibodies (ANA) are detectable in approximately 95% of cases, and disease‑specific autoantibodies, such as anti‑topoisomerase I and anti‑RNA polymerase III, typically seen in diffuse cutaneous SSc, and anti‑centromere antibodies associated with limited cutaneous SSc, are commonly present [5]. In a large retrospective EUSTAR cohort of 5,378 patients, 99.8% had detectable ANA or a history of RP [6]. In contrast, our patient initially lacked both RP and any autoantibodies; however, he later seroconverted and was found to be positive for anti‑RNA polymerase III.

Nailfold capillaroscopy is a crucial, noninvasive diagnostic tool in SSc, enabling direct visualization of microvascular changes that are characteristic of the disease. In SSc, distinctive nailfold capillaroscopic abnormalities are observed in most patients. These include enlarged capillary loops, micro‑hemorrhages, regions devoid of capillaries (avascular zones), architectural disorganization of the capillary bed, and evidence of new vessel formation [7]. Our patient demonstrated classic nailfold capillaroscopic findings consistent with SSc. Importantly, this non-invasive technique not only confirmed the diagnosis in a seronegative individual, where standard autoantibody assays were initially negative, but also effectively excluded mimicking conditions. Moreover, capillaroscopy facilitates very early detection of SSc, enabling the timely initiation of treatment before more overt manifestations arise.

Cardiac involvement in SSc is relatively common, with prevalence estimates ranging from 15% to 35% [8]. It manifests as myocardial damage, fibrosis of the conduction system, pericardial disease, arrhythmias, and ischemic complications [9]. Echocardiography detects pericardial effusion in 41% of SSc cases; however, clinically significant pericardial effusion is present in 5%-16% of patients with SSc, though tamponade is rare and carries a poor prognosis [8]. In our patient, given the rapid deterioration from baseline, tamponade secondary to the pericardial effusion was diagnosed. Prompt intervention was essential for a favorable prognosis.

## Conclusions

Diagnosing SSc remains challenging even for experienced clinicians and becomes particularly difficult in seronegative cases. In such patients, nailfold capillaroscopy proves invaluable, aiding in diagnostic confirmation and effectively excluding mimics of SSc. Cardiac involvement, while common in SSc, rarely progresses to tamponade; however, when it does, prompt recognition and intervention substantially reduce morbidity and mortality, improving patient outcomes.
